# Non-Thermal and Mild Thermal Technologies for Table Egg Shell Surface Decontamination: Microbial Efficacy, Egg Quality, and Industrial Considerations

**DOI:** 10.3390/microorganisms14020442

**Published:** 2026-02-12

**Authors:** Izadora Martina de Freitas Meireles, Wilson José Fernandes Lemos Junior, Amanda Mattos Dias-Martins, Marco Antônio Pereira da Silva, Claudio Cipolat-Gotet, Leandro Pereira Cappato

**Affiliations:** 1 Instituto Federal Goiano, Rio Verde 75901-970, GO, Brazilleandro.cappato@ifgoiano.edu.br (L.P.C.); 2 Department of Veterinary Sciences, University of Parma, 43126 Parma, Italy; claudio.cipolatgotet@unipr.it

**Keywords:** ozonation, pulsed light, cold plasma, catalytic ionization, ultraviolet light

## Abstract

Microbial contamination of table eggs remains an important food safety concern, largely due to the presence of *Salmonella* spp. on eggshell surfaces and the potential for cross-contamination along the collection, grading, and packing chain. Conventional sanitation practices, including chlorinated-water washing, can reduce surface microbial loads but may also present limitations related to cuticle alteration, process variability, water use, and the risk of recontamination when operational conditions are not tightly controlled. This review synthesizes evidence on non-thermal and selected mild thermal technologies for the surface decontamination of intact table eggs, including ultraviolet-C (UV-C) irradiation, pulsed light, ozone-based treatments (gas and microbubble systems), non-thermal plasma, plasma-activated water, and gas-phase hydroxyl radical processes. For each approach, antimicrobial performance is discussed alongside effects on eggshell integrity, cuticle preservation, and key quality indicators (e.g., Haugh unit, albumen pH, yolk color, and shell strength). Particular attention is given to industrial constraints that influence real-world performance, such as treatment uniformity and shading effects, humidity dependence, line speed, equipment integration, and validation criteria. A shared limitation of surface treatments is their inability to inactivate pathogens that have penetrated shell membranes or contaminated egg contents, underscoring the need to align technology selection with the targeted hazard and the regulatory context. Thus, available data indicate that non-thermal technologies can contribute to reducing eggshell contamination when properly optimized, although broader implementation will depend on standardized operating parameters, robust process validation, and regulatory acceptance within existing egg processing systems.

## 1. Introduction

According to the Food and Agriculture Organization of the United Nations (FAO), global egg production reached approximately 100 million tonnes in 2024, with hen eggs accounting for about 94% of total output [1]. Eggs represent one of the most widely produced and consumed animal-source foods worldwide, reflecting their economic relevance and role in food security. The scale of global production and trade highlights the importance of efficient and reliable safety strategies throughout the egg supply chain, particularly for maintaining quality and reducing microbiological risks in diverse production systems.

Eggs are widely consumed across all regions of the world due to their high nutritional value, affordability, and digestibility. They provide essential macro- and micronutrients as well as bioactive compounds with recognized health benefits, which contributes to their central role in human diets and reinforce their importance within global food systems [2]. Although concerns regarding cholesterol intake were previously raised, epidemiological evidence indicates no significant association between moderate egg consumption and increased cardiovascular disease risk [3]. Given their broad consumption across different population groups, ensuring the microbiological safety of table eggs remains a relevant public health priority.

Specifically, a previous study reported a 12% lower risk of stroke among individuals with higher egg intake (approximately one egg per day) compared to those with lower consumption, while no significant association was found between egg intake and CHD. This has reinforced their role as a high-quality dietary component. In addition to their nutrient content, eggs contain bioactive compounds that have been associated with potential health-promoting effects, including the modulation and prevention of certain diseases [2].

Despite their nutritional benefits, eggs may be exposed to contamination through different routes, with microbiological hazards, particularly *Salmonella* spp., representing the main food safety concern. Chemical contaminants may also occur depending on environmental exposure and agricultural practices, although their occurrence and relevance vary across production systems and regions [4]. The integrity of the cuticle and shell membranes is essential for limiting microbial penetration; therefore, surface decontamination strategies must balance microbial reduction with preservation of these natural protective barriers.

Egg contamination may occur either through surface exposure after oviposition or through internal contamination, including transovarian transmission. While post-harvest decontamination technologies primarily target microorganisms present on the eggshell surface, pathogens that have penetrated the shell membranes or contaminated the egg contents are not inactivated by surface treatments. This limitation is particularly relevant for *Salmonella enterica* subsp. *enterica* serovar Enteritidis, which remains a major regulatory concern. Consequently, the technologies discussed in this review are evaluated mainly in the context of eggshell surface decontamination of intact table eggs.

A deeper understanding of the egg’s structural complexity underscores the importance of preserving its natural defenses. The yolk, formed in the ovarian follicle and enveloped by the vitelline membrane after ovulation, serves as the primary nutrient source. The albumen, or egg white, is protein-rich and contains antimicrobial components as well as the chalazae fibrous, mucin-based structures that stabilize the yolk. The shell membranes, situated between the albumen and the shell, are composed of collagen and ovokeratin, cross-linked with disulfide and lysine bonds, which enhance their mechanical resilience. These membranes remain largely intact except in the region where the air cell develops [5].

The eggshell itself is a rigid matrix predominantly formed from calcium carbonate, deposited onto a proteinaceous scaffold. It is porous, enabling gas exchange while providing structural protection. The final outer layer, the cuticle, is rich in polypeptides and functions as the egg’s first barrier against microbial invasion. The complete formation of an egg from follicular development to oviposition takes approximately 8 to 10 days, with the majority of structural deposition occurring over a 24 h period in the oviduct [6,7].

Yolk stability is further influenced by lipid oxidation processes, particularly during storage. The presence of natural antioxidants such as carotenoids contributes to the maintenance of yolk quality [8]. Together, the shell, cuticle, and albumen form an integrated defense system against microbial threats. This emphasizes the relevance of hygienic and decontamination techniques that can enhance safety while maintaining the physical and functional integrity of the egg.

The egg industry has undergone significant changes driven by food safety requirements, consumer expectations, and evolving production practices. These factors have influenced both processing strategies and market preferences, emphasizing the need to evaluate technological approaches that support egg safety while maintaining product quality [9].

Eggs represent a potential source of microbial contamination, making the adoption of effective decontamination techniques crucial for preventing spoilage and preserving quality. Several non-thermal surface decontamination methods, such as ozone treatment, ultraviolet (UV) light, and cold plasma, have been widely studied as promising alternatives for the surface sanitation of intact eggs [10]. These approaches stand out due to their antimicrobial efficacy while maintaining the sensory and functional properties of eggs [11].

The egg industry is navigating a landscape marked by evolving demands related to animal welfare, environmental sustainability, and concerns surrounding human health and food safety. In response, producers have diversified their offerings and adapted production practices to better align with these expectations. A notable trend includes regulatory changes aimed at improving hygiene and product quality. For instance, in Brazil, Ordinance SDA No. 612 of 6 July 2022 requires the washing of dirty, non-cracked shell eggs and those intended for industrial processing; dirty eggs are defined as eggs with foreign material on the shell surface, including feces, soil, or egg yolk [12]. For intact eggs not intended for industrial use, washing remains optional. This regulation reflects a broader shift toward enhancing food safety and aligning with consumer concerns [12]. This process, regulated by the ordinance, involves the use of water at specific temperatures combined with approved sanitizers and is effective in reducing microbial loads. It is important to highlight that regulatory approaches to egg washing and sanitation vary considerably among countries, which directly influences the feasibility and role of alternative decontamination technologies within different egg processing systems [13].

However, conventional washing poses challenges related to high water consumption, effluent treatment requirements, and elevated operational costs, making it a less sustainable option. In contrast, innovative technologies, such as gaseous ozone treatment, offer a promising alternative for egg decontamination, significantly reducing microbial loads on eggshell surfaces. Additionally, ozone treatment eliminates the need for water and does not generate industrial waste, positioning itself as a sustainable alternative for the egg industry by ensuring microbiological safety while minimizing environmental impact [14].

This review aims to examine non-thermal and mild thermal technologies applied to the surface decontamination of intact table eggs, discussing their antimicrobial efficacy, effects on eggshell integrity and egg quality parameters, as well as key limitations affecting industrial implementation.

## 2. Microorganisms of Interest in Eggs

**Figure 1:**
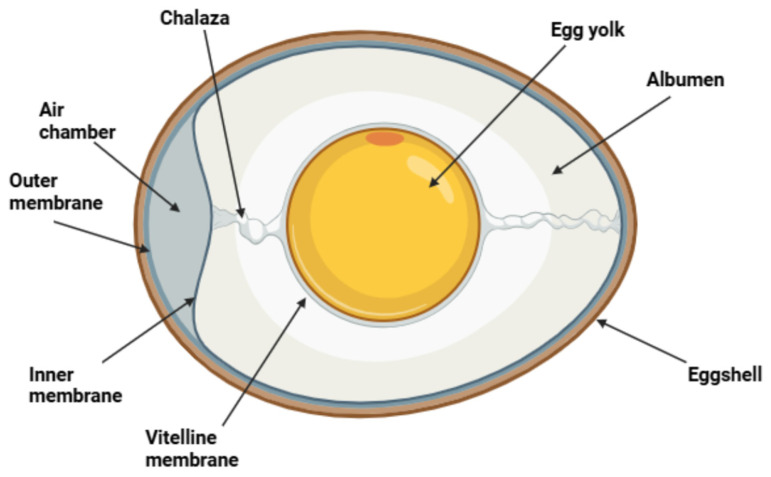
Schematic representation of the main structural components of a shell egg, including the eggshell, cuticle, shell membranes, albumen, yolk, and air cell. The figure was created using BioRender.

Eggs may be contaminated by a diverse microbiota originating from the production environment, handling practices, and post-laying exposure. Figure 1 illustrates the egg’s physiology, helping to explain why the eggshell is highly susceptible to microbial penetration due to its porous structure and the physiological conditions present at the time of oviposition. Gram-negative bacteria are frequently detected on eggshell surfaces and may exhibit an enhanced ability to migrate through shell pores under favorable conditions, particularly when temperature gradients occur immediately after oviposition [15]. The microorganisms most commonly associated with eggshell contamination include genera such as *Pseudomonas*, *Proteus*, *Escherichia*, *Serratia*, *Aeromonas*, *Citrobacter*, *Salmonella*, *Micrococcus*, *Staphylococcus*, and *Bacillus*, many of which are primarily linked to environmental exposure, fecal material, and post-laying handling conditions rather than intrinsic egg contamination [16,17].

From a food safety perspective, *Salmonella* spp. remain the most relevant pathogens associated with eggs and constitute the main target of regulatory control programs worldwide. In particular, *S.* Enteritidis is of concern due to its ability to contaminate eggshells and, in a smaller proportion of cases, egg contents, thereby posing a risk to consumers of raw or undercooked eggs [17,18]. Other microorganisms, including *Campylobacter*, *Listeria monocytogenes*, *Escherichia coli*, and *Staphylococcus aureus*, may be detected on eggshells but are more commonly associated with environmental contamination, cross-contamination during handling, or temperature abuse during storage rather than egg-specific foodborne outbreaks [19,20].

The susceptibility of freshly laid eggs to microbial ingress is strongly influenced by physicochemical factors. Eggs are laid at approximately 42 °C, and subsequent cooling creates negative pressure that may facilitate bacterial penetration through shell pores, especially when moisture is present on the shell surface [16]. This phenomenon highlights the importance of controlling environmental conditions immediately after laying and during early handling stages [21].

The prevalence of non-typhoidal *Salmonella* in commercial egg production has been documented in grading and packing facilities. In a survey involving 16,800 eggs collected from 60 commercial farms, *Salmonella* spp. were detected in 18.3% of eggshell samples and 20% of egg contents, with higher prevalence observed in flocks older than 80 weeks [22]. Age-related increases in contamination may be linked to changes in eggshell quality, immune status of laying hens, and cumulative environmental exposure over the production cycle [17].

Other microorganisms associated with environmental contamination have also been reported on eggshell surfaces. *Staphylococcus aureus* may be introduced during egg passage through the cloaca or through post-laying handling and storage, with its significance primarily related to inadequate hygiene and temperature control rather than intrinsic egg contamination [20]. Similarly, *Enterococcus* species, particularly *Enterococcus faecalis* and *Enterococcus faecium*, are frequently isolated from eggshells and are widely regarded as indicators of environmental contamination and hygienic conditions in egg production systems [16]. Although generally not primary egg-associated pathogens, these organisms raise concerns due to their opportunistic nature and increasing antimicrobial resistance.

High-throughput sequencing studies further indicate that the eggshell-associated microbiota is dynamic and strongly shaped by external factors such as season, handling sector, and post-lay exposure. Using full-length 16S rRNA sequencing, Gong et al. [23] reported that Firmicutes and Proteobacteria were the dominant phyla detected across hen egg samples, while *Staphylococcus*, *Acinetobacter*, *Aerococcus*, *Psychrobacter*, and *Lactobacillus* were among the most abundant genera on eggshell surfaces. Importantly, the dominant genera on farm eggshells varied across seasons, with a higher relative abundance of *Staphylococcus* observed in warmer periods, supporting the concept that temperature and humidity influence the microbial deposition and persistence on the shell. In market-derived samples, Gong et al. [23] also observed clear differences between the shell surface and egg contents, with *Staphylococcus* remaining frequent on shells, whereas *Pseudomonas* was more abundant in contents, reinforcing the need to distinguish surface contamination patterns from the microbiota detected internally. In addition, potential pathogenic taxa (e.g., *Klebsiella* spp. and *Escherichia*/*Shigella*) were detected, highlighting that eggs may carry opportunistic or hygiene-indicator organisms depending on production and distribution conditions.

## 3. Surface Decontamination of Eggs: Current Challenges and Emerging Solutions

Surface decontamination of shell eggs remains a critical control step for food safety, primarily due to the frequent detection of pathogens such as *Salmonella* spp. and *Escherichia coli* on eggshell surfaces. Conventional egg washing is widely applied in several countries to reduce surface microbial loads; however, this practice presents well-documented limitations related to microbiological efficacy, shell integrity, and process control [24,25]. One of the main concerns is the potential damage to the eggshell cuticle, which functions as the first physical barrier against microbial penetration. Exposure to detergents, mechanical brushing, or abrasive agents may partially remove or disrupt this layer, thereby increasing the risk of trans-shell contamination during subsequent handling and storage.

In addition, inadequate control of wash-water temperature can generate pressure differentials between the egg interior and the external environment, favoring bacterial ingress through shell pores. Reuse of wash water or insufficient sanitation of washing systems may further promote cross-contamination between eggs, while repeated washing has been associated with changes in shell appearance and, in some cases, reduced mechanical strength. Owing to these challenges, egg washing practices are regulated differently across regions, with mandatory washing in North America and restrictions or prohibitions in parts of Europe, reflecting distinct risk management strategies.

**Figure 2:**
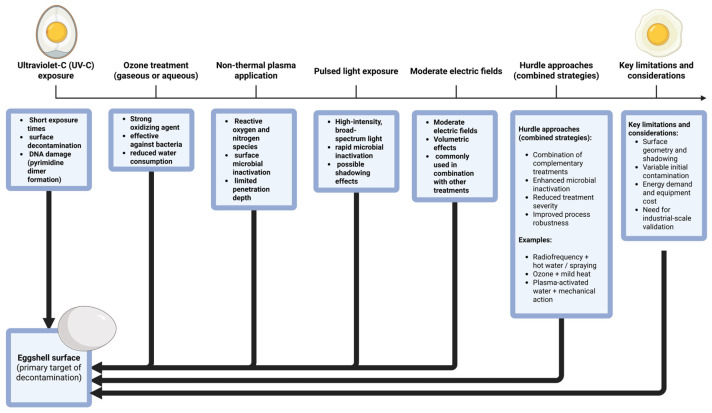
Overview of emerging technologies and hurdle approaches for eggshell surface decontamination. The figure was created using BioRender.

To address the limitations of conventional washing, a range of alternative surface decontamination technologies has been investigated. These approaches include ultraviolet-C (UV-C) irradiation, pulsed light (PL), gaseous ozone and ozone microbubbles, cold plasma, plasma-activated water (PAW), moderate electric fields (MEFs), hot air treatment, gas-phase hydroxyl radicals, and infrared (IR) irradiation [26]. Importantly, these technologies are designed for surface decontamination +d should be clearly distinguished from internal pasteurization methods, such as radiofrequency (RF) heating, which aim to inactivate pathogens that have penetrated the shell membrane. Conflating these fundamentally different objectives may lead to misinterpretation of their effectiveness and practical applicability (Figure 2).

Non-thermal surface treatments have attracted particular attention because they can reduce microbial contamination without relying on chemical disinfectants or prolonged water contact. Technologies such as UV-C irradiation, ozone exposure, cold plasma, and PAW have demonstrated the ability to inactivate a broad spectrum of surface-associated microorganisms while largely preserving internal egg quality. Nevertheless, each approach presents specific constraints, including dependence on surface geometry, shading effects, humidity requirements, formation of reactive by-products, and variability in achieved log reductions.

Despite encouraging laboratory-scale results, industrial implementation of these technologies remains limited. Challenges include the lack of standardized processing parameters, uncertainties regarding reproducibility under commercial conditions, capital investment costs, and compatibility with existing grading and packing lines. Moreover, regulatory acceptance varies between jurisdictions and often lags behind technological development.

The following sections therefore provide a structured overview of selected surface decontamination technologies, namely Plasma-Activated Water (PAW), Moderate Electric Fields (MEF), ozone-based treatments, Cold Plasma, Hot Air, UV-C irradiation, gas-phase hydroxyl radicals, Pulsed Light (PL), and Infrared Irradiation (IR). In addition, radiofrequency (RF) heating is included as an internal pasteurization approach, discussed separately to provide a contextual comparison with surface-based technologies. Emphasis is placed on mechanisms of action, reported antimicrobial efficacy, effects on shell and internal egg quality, and current limitations for industrial application.

The main surface decontamination technologies investigated for shell eggs differ substantially in their underlying physical and chemical mechanisms, as well as in their potential effects on egg quality. A conceptual overview of the mechanisms of action against microorganisms and the reported impacts on food attributes is summarized in Table 1.

### 3.1. Plasma Activated Water

PAW is an innovative technology that utilizes plasma an ionized state of matter composed of ions, electrons, and free radicals to alter the properties of water, enhancing its reactivity. During this process, electric fields, ultraviolet photons, reactive oxygen species (ROS), and reactive nitrogen-oxygen species are generated, all of which serve as potent antimicrobial agents. Although these reactive substances are relatively unstable and degrade rapidly, they offer the advantage of not leaving behind chemical residues, positioning PAW as a safe and environmentally friendly solution for both human health and the ecosystem [49].

In a study exploring its application for inactivating *Salmonella* Enteritidis on eggs, significant microbial reductions were observed reaching up to 5 log CFU/egg under optimized conditions [50]. The effectiveness of PAW was influenced by several operational parameters, including the number of plasma jets, the volume of water treated, and exposure time. The antimicrobial action was primarily attributed to the generation of reactive oxygen and nitrogen species during plasma activation [50]. These short-lived compounds are known for their potent biological activity, and because they degrade quickly, PAW does not leave chemical residues, making it a promising option for safe and sustainable egg decontamination.

In a comparative study, PAW achieved microbial inactivation levels on par with quaternary ammonium compounds (QA) compounds, reducing *Klebsiella michiganensis* by more than 5 log CFU/egg when eggs were massaged during treatment [51]. While simple submersion in PAW did not yield comparable reductions, the addition of mild mechanical action significantly enhanced sanitization efficiency. Importantly, PAW-treated eggs retained better cuticle integrity, as evidenced by colorimetric analysis, which showed significantly lower ΔE values compared to QA-treated counterparts, indicating minimal cuticle loss [51]. Mechanical testing confirmed that PAW did not compromise shell strength across various egg orientations. Furthermore, residual microbial counts in used PAW were below detectable limits, suggesting its ability to prevent cross-contamination during washing cycles.

Another study showed that both PAW and plasma-activated hydrogen peroxide (PAHP) effectively reduced *S.* Enteritidis, *Campylobacter jejuni*, and *S. aureus* on eggshells. PAHP achieved greater reductions, reaching up to 3 log CFU/egg at a 150 s treatment. Increased reactive species and a lower pH enhanced antimicrobial activity. DNA/RNA and protein leakage confirmed cell damage. PAHP maintained egg freshness but slightly reduced shell strength and cuticle integrity during storage [47].

A study investigated PAW conditions for microbial inactivation at egg-washing temperatures. Using response surface methodology, two optimal settings reduced *Salmonella enterica* subsp. *enterica* serovar Typhimurium by over 6 logs. Mixing and storing PAW up to 25 min did not compromise antimicrobial efficacy, despite declines in nitrite/nitrate. The avirulent *S.* Typhimurium MHM112 behaved similarly to pathogenic strains, supporting its use as a surrogate in safety assessments [52].

This study demonstrates the efficacy of PAW as a nonthermal, eco-friendly sanitizer for *Salmonella* Typhimurium on fresh produce and eggs. It highlights PAW’s comparable performance to chlorine while preserving food quality attributes such as color and pH. Nitric oxide emerged as the primary antimicrobial agent, supported by scavenger assays and membrane damage analysis. Both tap and deionized water-based PAW showed similar effectiveness, enhancing their practical application in the food industry [49].

Therefore, as an environmentally friendly technology that leaves no chemical residues, PAW holds significant potential for large-scale applications in the food industry, providing a sustainable and efficient solution for egg decontamination.

### 3.2. Moderate Electric Field (MEF)

MEF is an emerging decontamination technique that utilizes low-intensity electrical pulses to modify the permeability of microbial cell membranes, thereby enhancing microbial inactivation. When combined with ozonation, this synergistic approach improves pathogen reduction by leveraging the antimicrobial properties of ozone for disinfection.

In a study, MEF pretreatment was combined with ozonation to improve microbial inactivation in shell eggs, specifically targeting *Escherichia coli* K12 as a surrogate for *Salmonella* [53]. The application of MEF during water bath treatment significantly enhanced bacterial inactivation, achieving an additional reduction of approximately 0.94 log CFU/g compared to conventional heating, despite operating at slightly lower temperatures. This suggests that MEF facilitates cell membrane permeabilization, likely due to electroporation, enhancing microbial susceptibility to subsequent ozone treatment. Importantly, the improved efficacy of MEF allows for the possibility of lowering the thermal intensity of the process, potentially preserving the functional and sensory quality of eggs [53].

Although the antimicrobial effects of MEF are often attributed to electroporation phenomena and enhanced membrane permeability, its impact on egg quality particularly on egg white proteins has received increasing attention. A detailed investigation by Joeres et al. [54] demonstrated that ohmic heating under MEF conditions significantly altered the thermal denaturation behavior of egg white proteins compared to conventional heating. Using egg white protein systems heated from 25 to 85 °C, the authors showed that MEF interfered with intermolecular β-sheet formation, reduced hydrophobic interactions, and resulted in lower levels of ovalbumin denaturation. These structural effects were associated with more open and porous gel networks, as observed by scanning electron microscopy.

From an egg safety perspective, these findings are highly relevant because they indicate that MEF can modify protein unfolding and aggregation pathways without requiring prolonged exposure times or excessive thermal loads. This suggests that MEF-assisted treatments may allow microbial inactivation targets to be achieved while mitigating adverse effects on egg internal quality, such as excessive albumen thickening or loss of functional properties.

### 3.3. Ozone

Ozone is an allotropic form of oxygen (O_2_) consisting of three oxygen atoms, and it is characterized as a colorless gas with low stability due to its tendency to rapidly decompose into O_2_ [55]. Known for its high reactivity, ozone (O_3_) serves as a potent antimicrobial agent and is widely used in the food industry for decontaminating surfaces, water, and food products. In 2001, the FDA officially approved the use of ozone as an antimicrobial agent for food, recognizing its efficacy and safety. Ozone offers several advantages, such as low toxicity and ease of handling, and it decomposes spontaneously into non-toxic oxygen, generating virtually no residue [56].

In both the gaseous and aqueous phases, ozone is effective against a broad spectrum of microorganisms, including bacteria, fungi, yeasts, parasites, and viruses. Relatively low ozone concentrations and short exposure times are often sufficient to inactivate a wide range of pathogens. However, ozone’s effectiveness is maximized in systems that are free of oxidizable organic substances, as the presence of such compounds can reduce its antimicrobial efficacy. The most common applications of ozone in the food industry include the decontamination of product surfaces and water treatments [32].

In a study on egg decontamination, *S.* Typhimurium was inoculated onto eggshells, which were then treated with ozone at a concentration of 38.8 ppm for periods ranging from 10 to 30 min. The reduction in *S.* Typhimurium on the shells ranged from 4.22 to 5.25 log CFU/g, depending on the treatment time, which was significantly lower compared to the control group that presented 6.18 log CFU/g. Ozone treatment for 30 min resulted in a reduction of approximately 2 log CFU/egg, with this efficacy being maintained during storage. Moreover, the physical and chemical characteristics of the eggs, such as the Haugh unit, yolk color, pH of the white and yolk, foaming capacity, foam stability, and development of lipid oxidation, showed no significant differences in comparison to the control group [57].

An innovative technique involving ozone is microbubble formation, which presents an alternative to immersion in ozonated water. The application of ozone-combined microbubble water (OMB) for the decontamination of *Salmonella* Enteritidis on eggshells was investigated by Lin et al. [58]. OMB was generated by injecting ozone into a Nikuni microbubble system, applied in 10 L of water. After activation times of 5, 10, or 20 min, the eggs were washed for either 30 or 60 s. The greatest reduction observed was 5.19 log CFU/egg with a 20-min activation time and a 60-s washing period. After 15 days of storage at room temperature, the eggs treated with OMB showed sensory quality comparable to unwashed eggs, demonstrating the potential of this technique for large-scale application in the food industry [58].

In another study, ozone treatment enhanced the texture (hardness, chewiness) and water-holding capacity of heat-induced chicken egg yolk gels, peaking at 20 min of exposure [59]. Moderate oxidation promoted disulfide bond formation by reducing free sulfhydryl groups, stabilizing the gel network. Low-field NMR and SEM analyses showed improved water retention and a more ordered microstructure after ozonation [59]. Prolonged exposure (30–40 min) led to overoxidation, reducing gel performance and increasing cooking loss.

### 3.4. Cold Plasma (CP)

Cold plasma (CP) is a non-thermal, chemical-free, and environmentally friendly technology that has gained attention for its ability to effectively inactivate microorganisms, positioning it as a promising alternative in food safety. Cold plasma is generated by exposing a gaseous medium to an energy field, which leads to partial or complete ionization. This plasma contains a variety of charged particles, free electrons, and reactive species. These species can be classified into long-lived reactive species, such as hydrogen ions (H^+^), nitrite (NO_2_^−^), nitrate (NO_3_^−^), hydrogen peroxide (H_2_O_2_), and ozone (O_3_), and short-lived reactive species, such as reactive oxygen species (ROS), reactive nitrogen species (RNS), superoxide (O_2_), and hydrogen superoxide (HO_2_^−^) [36,60].

Several operating conditions were evaluated in a study using a cold plasma jet device to decontaminate chicken eggshells inoculated with *Escherichia coli* and *Salmonella enterica*. The variables studied included power levels (300–400 W), exposure times (20–60 s), distances between the emission nozzle and the shell (1–3 cm), air flow rates (30–35 L/min), and feed gases (nitrogen, air with 20–65% relative humidity, and helium–air mixtures). The results showed a significant reduction in microbial load, with a maximum reduction of 1.94 log (98.74%) for *E. coli* and 1.11 log (92.20%) for *Salmonella*, after 60 s of cold plasma treatment, at a 1 cm distance, with 400 W power, 35 L/min air flow, and 65% relative humidity. In addition to antimicrobial efficacy, cold plasma treatment did not cause significant changes in egg quality, nor in the chemical composition of the cuticle or shell coating, compared to untreated eggs. These results highlight the potential of cold plasma as an emerging, effective, non-chemical and non-thermal technique, offering a viable alternative to conventional egg washing methods, with the added advantage of not compromising the sensory and structural quality of the product [61].

The study by Lin et al. [62] represents the first attempt to evaluate the efficacy of a large-scale device using Non-Thermal Plasma (NTP) for egg decontamination. The NTP system was operated at 400 W (power), 30 slm (airflow rate), 5 and 10 eggs/min (conveyor belt speed), and 4 cm (distance between plasma jets and egg samples). A two-round treatment using the 400 W-30 slm-5 eggs/min-4 cm configuration achieved the highest reduction in *Salmonella* Enteritidis populations (3.57 log CFU/egg). The study also showed less surface damage to plasma-treated eggs compared to commercially washed eggs. Overall, NTP treatment maintained the freshness and sensory qualities of the eggs for up to 15 days of storage.

### 3.5. Hot Air

Hot air treatment is a thermal decontamination technique that uses air circulation at high temperatures to inactivate microorganisms present on the surface of foods, such as eggs. This approach has been shown to be effective in eliminating pathogens, such as *Salmonella* Enteritidis, without causing significant damage to the internal contents or sensory properties of the eggs. The technique can be adjusted to optimize process efficiency and minimize negative impacts, such as changes in egg flavor or texture. In one study, a device was developed to treat eggs using two jets of hot air at 600 °C, alternating with intervals of cold air. This treatment resulted in a reduction of up to 1.9 log in the *S.* Enteritidis load on the shells, without significantly affecting egg quality. The results indicate that hot air treatment may be an effective alternative to water pasteurization, widely used in the United States but banned in the European Union for grade A eggs. The technique has shown promise for decontaminating eggs without causing damage to the shell or internal components of the egg. Egg quality was evaluated both immediately after treatment and after 28 days of storage, with results indicating that there were no significant changes in quality parameters such as pH, albumen turbidity, shell color, and yolk index [37].

### 3.6. UV-C Irradiation

Ultraviolet-C (UV-C) irradiation has been extensively investigated as a physical, non-chemical approach for eggshell surface decontamination due to its well-defined antimicrobial mechanism and compatibility with industrial egg handling systems. UV-C radiation operates within the 200–280 nm wavelength range, which corresponds to the germicidal region of the ultraviolet spectrum. In this range, microbial inactivation occurs primarily through irreversible damage to nucleic acids, particularly via the formation of pyrimidine dimers that inhibit DNA replication and cell division [63]. This mechanism is especially relevant for surface-associated microorganisms on eggshells, where direct exposure to UV photons enables effective disruption of microbial viability without the use of water or chemical agents.

In the context of table eggs, the effectiveness of UV-C is influenced by factors inherent to the eggshell matrix, including surface curvature, porosity, and the presence of organic residues, which can create shadowed areas and limit uniform dose delivery [63]. Despite these constraints, short UV-C exposures integrated into grading and packing lines have demonstrated consistent reductions in naturally occurring eggshell microbiota while preserving shell integrity and internal egg quality. Consequently, UV-C irradiation is best positioned as a surface-focused intervention within a hurdle-based sanitation strategy rather than as a standalone lethality step for internally contaminated eggs.

Industrial-scale application of UV-C has demonstrated measurable microbial reductions under realistic processing conditions. In a commercial egg grading and packing system equipped with UV-C lamps (253.7 nm; 10 mW·cm^−2^), an exposure time of 7 s resulted in a reduction of approximately 0.85 log_10_ CFU/eggshell in total aerobic microorganisms on naturally contaminated table eggs [64]. Under the same conditions, reductions of >0.18 log_10_ CFU/eggshell for Enterobacteriaceae, >0.17 log_10_ CFU/eggshell for *Escherichia coli*, and 0.77 log_10_ CFU/eggshell for yeasts and molds were observed. Pathogenic microorganisms, including *Salmonella* spp., *Listeria monocytogenes*, and *Campylobacter* spp., were below the limit of quantification (<1.26 log_10_ CFU/eggshell) both before and after treatment, indicating low initial prevalence and no pathogen proliferation following irradiation [64].

These results confirm that short UV-C exposures applied in industrial settings can achieve consistent, although moderate, reductions of surface-associated microbiota. Nevertheless, UV-C efficacy is inherently constrained by eggshell surface geometry, shadowing effects, and the shielding of microorganisms within pores or organic residues, which limits the achievable log reduction under practical conditions.

From a quality perspective, UV-C irradiation offers advantages because it operates without added moisture or elevated temperatures, thereby preserving shell integrity and internal egg quality. However, as its action is restricted to exposed surfaces, UV-C irradiation should be regarded as a complementary intervention rather than a standalone lethality step, particularly in situations involving higher initial contamination levels.

### 3.7. Hydroxyl Radical in Gas Phase

The gas-phase hydroxyl radical (OH) process is an emerging decontamination technology with potential applications to food surfaces such as eggshells. This approach relies on the generation of highly reactive hydroxyl radicals through the decomposition of hydrogen peroxide (H_2_O_2_) in the presence of ozone (O_3_) and UV-C radiation. Owing to their strong oxidative capacity, these radicals disrupt microbial cell membranes and intracellular components, resulting in effective microbial inactivation.

A key advantage of this technology is its non-contact mode of action, which is particularly suitable for sensitive products such as eggs, where preservation of structural integrity is required. In a study evaluating the inactivation of *Salmonella* Enteritidis on eggshells, a treatment combining 2% (*v*/*v*) hydrogen peroxide, 20 ppm ozone, and a UV-C dose of 19 mJ/cm^2^ applied for 10 s achieved reductions exceeding 5 log CFU/egg, with *Salmonella* reduced to levels close to or below the limit of detection [65].

This treatment did not adversely affect internal egg quality parameters, including pH, Haugh unit, or yolk characteristics, and no negative effects were observed on the hatchability of fertilized eggs. Together, these results indicate that gas-phase hydroxyl radicals can provide high antimicrobial efficacy while maintaining egg quality, supporting their potential as a rapid, non-invasive surface decontamination approach for shell eggs [65].

### 3.8. Pulsed Light (PL) and Pulsed Ultraviolet Light (PUV)

Pulsed Light (PL) and Pulsed Ultraviolet Light (PUV) are advanced non-thermal decontamination technologies that utilize high-intensity, short-duration flashes of light to effectively inactivate microorganisms on food surfaces, such as eggshells. Both technologies rely on the emission of rapid and powerful light pulses, which provide an efficient method for microbial inactivation with minimal exposure time. The primary mechanism of microbial inactivation for both PL and PUV is the formation of thymine dimers in the DNA of microorganisms, which prevents their replication. Additionally, the pulses generate localized heating and microvibrations, which further contribute to the destruction of cell membranes, amplifying their germicidal effects.

A key difference between the two is that PL emits a broad spectrum of light (200–1100 nm), with about 40% of its energy in the ultraviolet (UV) range (100–400 nm). This broader wavelength range allows PL to interact with various microbial targets, such as DNA, cell membranes, and proteins. In contrast, PUV specifically targets the UV radiation range, which makes it more efficient in terms of germicidal efficacy per unit of energy. Both technologies have shown considerable promise in the decontamination of eggs. Studies have demonstrated that doses of 2.1 J/cm^2^ resulted in reductions of up to 5 log CFU/egg for pathogens like *Salmonella* and *Escherichia coli*, without significantly increasing shell temperature or compromising egg quality. At higher doses, such as 10.5 J/cm^2^, both PL and PUV treatments maintained shell integrity and did not cause microbial penetration into the internal egg content, ensuring the safety and quality of the eggs [43,66].

### 3.9. Infrared Irradiation (IR)

Infrared irradiation (IR) is a thermal decontamination technique based on the application of electromagnetic radiation, typically within the wavelength range of approximately 3 to 1000 µm, to induce rapid heating of exposed surfaces. In the context of shell eggs, IR primarily promotes surface heating of the eggshell without direct contact, which is advantageous for reducing microbial loads while limiting heat transfer to internal components. The mechanism of heating relies on the absorption of infrared energy by eggshell constituents and surface-associated moisture, followed by conversion into heat through molecular vibration. The effectiveness of IR treatment on eggs is influenced by factors such as radiation wavelength, emitter intensity, exposure time, shell color, surface moisture, and eggshell thickness, all of which affect heat absorption and distribution. Because infrared radiation exhibits limited penetration depth, its application is particularly suited for surface decontamination rather than internal pathogen inactivation. Common infrared emitters employed in food applications, including quartz lamps, ceramic emitters, and halogen lamps, differ in emission spectra and heating rates, which can influence temperature uniformity on the curved eggshell surface and must be carefully controlled to avoid localized overheating or quality degradation [46].

In the context of egg decontamination, infrared heating has been explored as a method to reduce microbial load on eggshells. A study investigating the application of IR on eggs inoculated with *Escherichia coli* ATCC 25922, a strain with heat resistance comparable to *Salmonella* Enteritidis, showed promising results. The eggs were exposed to IR at temperatures ranging from 180 to 350 °C, and a temperature of 250 °C for 110 s resulted in a 3.37 log reduction in microbial load. Importantly, this treatment did not cause albumen denaturation or significant changes in egg quality parameters such as yolk index, Haugh unit, albumen pH, or foaming capacity. It suggested that infrared heating could be a highly effective and non-invasive method for surface decontamination of eggs, preserving both the sensory and functional quality of the product while improving food safety [45]. This makes IR a promising technique for use in the food industry.

### 3.10. Radiofrequency (RF)

Radiofrequency (RF) heating is a dielectric thermal technology in which eggs are exposed to electromagnetic fields (typically 3 kHz–300 MHz), enabling rapid volumetric heating of internal components. In shell eggs, RF differs from surface decontamination approaches because energy absorption and heat generation occur primarily in the yolk/albumen, supporting its use as an internal pasteurization strategy targeting pathogens that may be present inside the egg rather than on the shell. A central rationale for RF-based pasteurization is that it can raise internal temperatures faster than conventional hot water processes that rely on conduction from the shell inward, thereby reducing the overall time needed to reach lethal conditions while limiting quality losses when parameters are properly controlled.

In the most recent RF with heat configuration, Bermudez-Aguirre et al. [39] inoculated eggs internally with *Salmonella* Typhimurium (ca 10^5^ CFU/egg) and applied RF at 40.68 MHz and 35 W for 4.5 min, followed by a hot water spray at 56.7 °C for 20 min (total process time 24.5 min). This treatment achieved approximately 5-log reduction (reported as −5.0 to −5.2 log, depending on cooling approach), with no sublethally injured cells detected and no recovery during 5 days at 7 °C (limit of detection <1 log CFU/g). Importantly, most egg quality attributes were similar to controls (e.g., Haugh unit, yolk index, shell strength, yolk/albumen pH), although albumen turbidity/absorbance increased after processing, indicating some protein structural change under the applied conditions.

Earlier work by Yang and Geveke [67] demonstrated the same principle using RF as a rapid internal heating step combined with a controlled external thermal step to complete pasteurization. They reported that the time needed to achieve 5-log reductions in *S.* Typhimurium was 19.5 min for RF + hot water immersion (RF/HWI) and 24.5 min for RF + hot water spraying (RF/HWS). In that study, yolk index was largely unaffected, while Haugh unit and albumen turbidity increased with longer treatment time, reinforcing that microbial lethality and product quality are tightly linked to the cumulative thermal exposure profile, even when RF accelerates come-up times.

Conceptually, RF + heat belongs to the broader family of hybrid thermal strategies where a fast “come-up” mechanism is paired with a steadier holding step to control lethality and quality. This is similar in logic (though not in mechanism) to how ultrasound is often discussed in food microbiology: ultrasound alone is typically insufficient in real foods, but combinations with heat/pressure improve efficacy and energy efficiency highlighting why integrated processes such as RF with external heating are attractive for sensitive matrices like eggs [68].

Although Table 2 summarizes microbial reductions and quality-related outcomes, a direct comparison of treatment efficiency across technologies should be interpreted with caution. The reviewed approaches are based on fundamentally different mechanisms of action and are reported using heterogeneous processing metrics, including exposure time, radiation dose, electrical power, oxidant concentration, or temperature. As a result, similar log reductions may reflect markedly different process intensities and operational constraints. Technology-specific processing conditions and limitations are therefore discussed within the corresponding subsections to support a context-dependent interpretation of antimicrobial efficacy.

## 4. Perspectives for the Use of Emerging Technologies in the Egg Chain and Main Challenges

The integration of alternative technologies into egg processing has shown potential to enhance food safety while responding to increasing environmental and regulatory concerns. Approaches such as gaseous ozone, non-thermal plasma, pulsed light, and ultraviolet-C (UV-C) radiation have demonstrated the capacity to reduce microbial loads on eggshell surfaces under controlled conditions, without major adverse effects on egg quality when appropriately applied. Consequently, these technologies have been proposed as alternatives or complements to conventional sanitization practices, particularly those based on chlorinated water, which are associated with high water consumption, chemical inputs, and effluent management requirements.

Across the technologies reviewed, it is evident that each method presents distinct advantages but also inherent limitations that influence its stated efficacy and industrial applicability. These limitations have been addressed within the respective sections of this review and include factors such as dependence on surface geometry and shadowing effects for radiation-based treatments, variability in reactive species generation for plasma- and ozone-based systems, and sensitivity to operational parameters such as exposure time, humidity, and organic load. The recognition of these constraints is essential for interpreting reported log reductions and for avoiding overestimation of performance under commercial conditions.

Although Radiant Catalytic Ionization (RCI) has demonstrated antimicrobial potential in air treatment and general surface sanitation contexts, its application to eggshell decontamination remains insufficiently characterized [34,71]. Evidence specific to egg systems is currently limited, particularly regarding treatment uniformity on curved shell surfaces, potential effects on cuticle integrity, and validation under industrial processing conditions. Given the wide range of decontamination technologies already addressed in this review and the comparatively limited body of egg-focused data available for RCI, this technology was not included as a standalone section.

Where reliable data are available, the integration of hurdle technologies emerges as a promising strategy to overcome the limitations associated with single decontamination interventions. Several studies indicate that combining emerging non-thermal methods with mild thermal or physicochemical steps can enhance microbial inactivation while reducing the intensity required for each individual treatment. Examples discussed in this review include the association of radiofrequency heating with hot water or hot water spraying, moderate electric fields combined with ozonation, and plasma-activated water coupled with mechanical action. Such combinations allow higher and more reproducible log reductions to be achieved while minimizing adverse effects on eggshell integrity and internal quality.

From an industrial perspective, hurdle approaches may also improve process robustness by compensating for variability in surface contamination, shell morphology, and organic matter distribution. However, systematic evaluations of combined treatments under industrial conditions remain limited. Future research should therefore prioritize the optimization of processing sequences, the validation of synergistic or additive effects, and the assessment of regulatory acceptance.

## 5. Conclusions

The transition toward alternative decontamination technologies in the egg industry reflects broader efforts to improve food safety while addressing efficiency and environmental considerations. This review summarizes current evidence on non-thermal and residue-free approaches, including ozone, pulsed light, ultraviolet-C radiation, non-thermal plasma, and plasma-activated water, with emphasis on their antimicrobial performance, effects on eggshell integrity, and implications for egg quality. Collectively, these technologies may reduce dependence on chemical disinfectants and water-intensive processes while preserving key functional and structural attributes of shell eggs.

However, large-scale implementation remains contingent on overcoming several challenges, including process standardization, economic constraints, and regulatory alignment. Future research should prioritize comparative assessments under industrially relevant conditions and the development of integrated processing concepts that balance microbial control with environmental performance. Policy incentives, coordinated research efforts, and collaboration between industry and regulatory bodies will be central to enabling informed decision-making. In this context, emerging decontamination technologies may contribute to the development of safer, more efficient, and environmentally responsible egg processing systems.

## Figures and Tables

**Table 1:** Decontamination methods and their mechanisms of action against microorganisms and food.

Decontamination Methods	Mechanism of Action on the Microorganism	Changes in Food	References
Non-thermal plasma (NTP).	Reactive oxygen species (ROS) and reactive nitrogen species (RNS) induce lipid peroxidation, damage proteins, and alter DNA, compromising cellular integrity and ultimately leading to cell death. These species have a more pronounced effect on Gram-negative bacteria, as their outer membrane is rich in lipopolysaccharides, which makes them more susceptible to oxidative damage.	NTP (Non-Thermal Plasma): May induce lipid peroxidation and protein denaturation in food, altering its sensory and nutritional properties.	[27,28]
Moderate Electric Field (MEF).	Moderate Electric Field (MEF) modifies cell permeability by creating pores in the membrane, facilitating the entry of antimicrobials. When combined with shear forces, it further amplifies membrane damage, thereby enhancing microbial inactivation.	MEF (Moderate Electric Field): Can affect the texture, pH, and structure of food proteins due to low-intensity pulses.	[29,30]
Ozone.	Ozone oxidizes membrane lipids, proteins, and nucleic acids, leading to cell membrane dysfunction and the inactivation of essential enzymes, thereby compromising cellular metabolism.	Ozone: Can lead to lipid peroxidation and protein oxidation, impacting the texture, flavor, color, and stability of bioactive compounds in food. It may also alter the pH and sensory attributes of food.	[31,32]
Radiant Catalytic Ionization (RCI).	Oxidizing agents induce lipid peroxidation, disrupting the cell membrane and increasing its permeability. Additionally, they damage proteins and nucleic acids, leading to enzyme inactivation and causing lethal mutations in DNA and RNA.	RCI (Radiant Catalytic Ionization): Generates reactive oxygen species (ROS) that oxidize lipids, proteins, and nucleic acids, thereby altering the texture, stability, and functional and sensory properties of food.	[33,34]
Cold Plasma.	The generation of reactive oxygen species (OH, O_3_, H_2_O_2_) and nitrogen species (NO, NO_2_, NO_3_) induces lipid peroxidation, compromising the cell membrane and increasing its permeability. Damage to bacterial DNA and RNA hinders replication and repair processes. Additionally, the reduction in pH (from 7.4 to 3.4) further enhances the antimicrobial effects.	Cold Plasma: Produces reactive oxygen species (ROS) and nitrogen species (RNS) that interact with lipids and proteins, causing lipid peroxidation, which may affect the texture and flavor of food.	[35,36]
Hot air.	Heat denatures membrane proteins and lipids, leading to rupture and increased permeability, which results in the loss of cellular integrity and intracellular leakage. It also denatures essential proteins and can induce breaks in DNA and RNA, thereby preventing replication and transcription.	Hot Air: Can reduce moisture, which affects softness and freshness, and may cause minor variations in pH and the color of the eggshell and albumen.	[37]
Radio frequency.	Radiofrequency (RF) energy disrupts cell membrane function, increasing permeability and causing leakage of essential intracellular components. This leads to protein denaturation and membrane destruction, ultimately inducing cell death. In addition to its thermal effects, RF energy can also damage DNA, inhibiting replication and interfering with metabolic reactions.	RF (Radiofrequency): Can denature proteins found in food, such as those in egg whites and yolks. It may also induce small variations in the pH of the food.	[38,39]
UV C irradiation.	UV-C radiation (200–280 nm) induces damage to microbial DNA by forming thymine dimers, which hinder replication and result in cell death. It also affects proteins and cell membranes, compromising structural integrity. UV-C is an effective method that does not generate chemical residues, making it a sustainable alternative for decontaminating foods such as eggs.	UV-C Radiation: Can cause slight changes in pH and color, promoting oxidation, but these effects are minimal when the radiation is controlled, thus preserving sensory quality and ensuring food safety.	[40,41]
Pulsed Light (PL).	UV-C radiation from pulsed light induces thymine dimer formation in DNA, disrupting replication and leading to cell death. It also physically damages cell membranes, increasing permeability and compromising cellular integrity. Although non-thermal, pulsed light can generate localized heat, which aids in the inactivation of more resistant structures, such as spores.	Pulsed Light (PL): Can alter the texture and color of food, particularly eggs and fruits, and cause lipid oxidation in fat-rich foods.	[42,43]
Pulsed Ultraviolet Light (PUV).	Pulsed ultraviolet light (PUV) operates by damaging DNA through the formation of thymine dimers (UV 100–400 nm) and causing cell membrane collapse via microvibrations and heating (UV 400–1100 nm). Additionally, it increases permeability and destabilizes intracellular proteins due to localized heating.	UVP (Pulsed Ultraviolet Light): Like UV-C, UVP can cause lipid oxidation, particularly in lipid-rich foods, which may affect the stability and flavor of the product. In some foods, UVP may cause small changes in pH and occasionally alter the structure of proteins.	[44]
Infrared Radiation (FIR).	Far-infrared radiation (FIR) inactivates microorganisms by elevating their internal temperature, leading to the denaturation of essential proteins, alteration of cell membrane permeability, and DNA damage. While effective against heat-sensitive microorganisms, FIR requires more intense conditions to effectively kill spores.	Far-Infrared Radiation (FIR): Preserves nutritional properties better, with less impact on vitamins and nutrients, and is a more sustainable technology, as it does not require large volumes of water.	[45,46]
Plasma Activated Water (PAW).	Plasma Activated Water (PAW) inactivates microorganisms by damaging the cell membrane through lipid peroxidation, oxidizing proteins and nucleic acids, and disrupting redox metabolism, leading to oxidative stress. Its low pH and high oxidation-reduction potential further intensify these effects, enhancing its antimicrobial action.	Plasma Activated Water (PAW): Can cause lipid peroxidation, affecting food stability and flavor, with slight alterations in pH, especially in foods that absorb acidic compounds. PAW maintains better preservation of nutritional properties compared to heat treatments, as it does not apply direct heat, thereby retaining vitamins and nutrients.	[47,48]

**Table 2:** Articles presenting eggshell decontamination using innovative technologies.

Decontamination Method	Target Microorganism	Observed Reductions	Impact on Egg Quality	Reference
Non-Thermal Plasma (NTP) on an industrial scale	*Salmonella* Enteritidis	Reduction of up to 3.57 log CFU/egg after two passes through NTP.	Non-Thermal Plasma (NTP) treatment did not significantly alter the sensory quality of the eggs and preserved freshness characteristics for up to 15 days.	[62]
Moderate Electric Field (MEF) in combination with Ozonation	*Escherichia coli* K12 (used as a substitute for *Salmonella*).	Inactivation of 0.94 log CFU/g compared to conventional heating.	The temperature was controlled to maintain the integrity of egg proteins.	[53]
Gaseous Ozone	*Salmonella* Typhimurium and *Pseudomonas aeruginosa*	A 4D reduction was achieved in 52.5 and 51.8 min at 75 ppm, and in 49.0 and 44.8 min at 110 ppm, for *Salmonella* Typhimurium and *Pseudomonas aeruginosa*, respectively	No significant quality differences were observed between ozonated and control eggs during storage.	[14]
Radiant Catalytic Ionization (RCI) and Ozonation	Three strains of Salmonella enterica (*S.* Enteritidis, *S. typhimurium*, and *S. virchow*).	Reduction of up to 3.54 log CFU/egg with RCI and up to 2.73 log CFU/egg with ozonation.	There were no details provided regarding sensory or physical quality.	[34]
Hot Air Treatment	*Salmonella* Enteritidis	Reduction of up to 1.9 log CFU/egg.	The treatment did not affect the pH, color, or shell resistance.	[37]
Ozonation and UV-C Irradiation	Mixture of three strains of *Salmonella* enterica.	Reduction of 2 log CFU/egg with UV-C; ozone was less effective.	UV-C treatment better preserved antioxidants and cholesterol levels in the yolk.	[69]
Ozone Microbubbles (OMB)	*Salmonella* Enteritidis	Maximum reduction of up to 5.19 log CFU/egg in 10 L of water.	No significant changes in sensory quality were observed after 15 days.	[58]
Hydroxyl Radical Process in Gas Phase	*Salmonella* Enteritidis	Reduction of more than 5 log CFU/egg.	The cuticle and integrity of the eggs were preserved.	[65]
Cold Plasma	*Salmonella* Enteritidis e *Escherichia coli*.	Up to 1.94 log reduction for *E. coli* and 1.11 log for *Salmonella*.	There were no significant changes in the chemical composition or physical quality of the eggs.	[61]
Radiofrequency combined with hot water (RF/HW)	*Salmonella* Typhimurium	>5 log reduction from initial 2.5 log CFU/mL; partial reduction from 6.5 log CFU/mL (to 0.7 log CFU/mL).	Physical quality was similar to untreated eggs, with a more cloudy albumen.	[67]
UV-C irradiation	Total aerobes, Enterobacteriaceae, *Escherichia coli*, yeasts and molds.	Up to 0.85 log CFU/egg reduction.	No specific information was provided.	[64]
Pulsed Light (PL)	*Escherichia coli* ATCC 8739	Up to 3.77 log CFU/egg reduction.	There was no significant impact on egg temperature, and internal properties remained stable for 4 weeks.	[66]
Far Infrared Radiation (FIR)	*Escherichia coli* ATCC 25922	3.37 log CFU/egg reduction.	No adverse effects were observed on internal quality.	[45]
Plasma Activated Water (PAW)	*Salmonella* Enteritidis ATCC 13076	5 log CFU/egg reduction in 60 s.	The treatment did not compromise sensory integrity or internal quality.	[50]
Pulsed Ultraviolet Light (PUV)	*Escherichia coli* K12-NSR e *Enterococcus faecium*.	Up to 4.54 log CFU/cm^2^ reduction for *E. coli*.	The temperature increase was insignificant, with no negative impact on internal quality.	[70]

## Data Availability

The original contributions presented in this study are included in the article. Further inquiries can be directed to the corresponding author.
